# Peroxiredoxin-Mediated Redox Regulation in Neurons: From Neurite Development to Degeneration

**DOI:** 10.3390/antiox15050604

**Published:** 2026-05-10

**Authors:** Gyuree Kim, Jaeyeon Lee, San Kwon, Eunbyul Yeom, Dong-Seok Lee

**Affiliations:** 1 BK21 FOUR KNU Creative BioResearch Group, School of Life Sciences, Kyungpook National University, Daegu 41566, Republic of Korea; gkellie23@knu.ac.kr (G.K.); leejydavid@knu.ac.kr (J.L.); rnjstks00@knu.ac.kr (S.K.); yeb@knu.ac.kr (E.Y.); 2 School of Life Sciences & Biotechnology, College of Natural Sciences, Kyungpook National University, Daegu 41566, Republic of Korea

**Keywords:** peroxiredoxin, redox signaling, neuronal differentiation, mitochondrial homeostasis, endoplasmic reticulum stress, neurodegenerative disease

## Abstract

In the nervous system, reactive oxygen species (ROS) serve essential roles in intracellular signaling, but their dysregulation can impair neuronal function and survival. Peroxiredoxins (Prdxs) have classically been regarded as antioxidant enzymes that scavenge peroxides, yet a growing body of evidence indicates that their roles in the brain extend beyond ROS removal. They are increasingly recognized as regulators of redox-dependent processes with isoform-specific roles. In this review, we discuss the functions of Prdxs in the brain from a broad cellular perspective, focusing on their roles in neuronal differentiation, mitochondrial and endoplasmic reticulum (ER) homeostasis, and major neurological disorders including Alzheimer’s disease, Parkinson’s disease, and ischemic stroke. Prdx isoforms show distinct condition-dependent functions regulated by localization, regulatory state, and cellular environment. Collectively, a broader view of Prdxs as dynamic modulators of neural cell biology may help us understand their coordinated integrate their roles in the coordinated regulation of redox-sensitive cellular processes. Clarifying the isoform-specific and cell-type-specific dependent mechanisms underlying their function will be essential in defining the roles of Prdxs in brain physiology and diseases and to evaluating their therapeutic potential.

## 1. Introduction

Reactive oxygen species (ROS) are indispensable signaling molecules in the nervous system, where precise redox regulation is required to maintain cellular function and homeostasis [1,2,3]. Because the brain is characterized by high oxygen consumption, abundant oxidizable lipids, and limited regenerative capacity, maintenance of redox balance is particularly critical in neural tissues [4,5,6]. Among the major thiol-dependent antioxidant systems, the Prdx family of enzymes plays a central role in peroxide metabolism and redox signaling [7,8]. Prdxs should not be regarded as a uniform group of interchangeable antioxidant enzymes. Instead, individual Prdx isoforms differ in catalytic mechanism, subcellular localization, tissue distribution, and biological function [9,10,11,12].

Mammalian cells express six Prdx isoforms, Prdx1–Prdx6, which are commonly classified according to their catalytic cysteine configuration. Prdx1–Prdx4 are typical 2-Cys Prdxs, Prdx5 is an atypical 2-Cys Prdx, and Prdx6 is a 1-Cys Prdx [13,14,15]. Prdx3 is primarily mitochondrial and is closely associated with mitochondrial peroxide removal and redox homeostasis. Prdx4 is localized mainly to the endoplasmic reticulum (ER) and secretory pathway, where it links peroxide metabolism to oxidative protein folding and ER proteostasis. Prdx5 is distributed across multiple compartments, including mitochondria, peroxisomes, cytosol, and nucleus, suggesting a broader role in compartmentalized redox regulation [16]. In contrast, Prdx6 is a 1-Cys Prdx with both peroxidase and phospholipase A2 activities, making it biochemically distinct from other Prdx family members [17].

In the brain, Prdx isoforms also show cell-type- and compartment-dependent patterns of expression. Previous studies have reported Prdx expression in neuronal populations as well as in glial cells, but the functional interpretation of each isoform must consider its cellular source [18,19]. This point is particularly important for Prdx6, which is predominantly associated with astrocytes in the central nervous system; therefore, its effects on neurons or microglia may often reflect astrocyte-mediated or glia-dependent mechanisms rather than direct neuron-autonomous actions [19,20]. In addition, mitochondrial Prdx3 and Prdx5, ER-localized Prdx4, and cytosolic Prdx1/2 contribute to distinct aspects of neural redox regulation, indicating that Prdx functions in the nervous system are shaped by both isoform identity and subcellular localization [21,22,23]. To provide a clearer framework for the isoform-specific discussion, the biochemical classification, major subcellular localization, and reported neural cell-type distribution of mammalian Prdx isoforms are summarized in Table 1.

This isoform-specific view is especially relevant in the brain, where redox regulation must be coordinated across multiple biological processes, including neuronal differentiation, organelle homeostasis, stress adaptation, and inflammatory responses [21,24]. Accumulating evidence indicates that Prdxs do more than simply eliminate excess peroxides; they can regulate redox-sensitive signaling pathways by controlling local H_2_O_2_ availability, participating in thiol-disulfide exchange, undergoing oligomerization or post-translational modification, and, under pathological conditions, acting as extracellular damage-associated molecular patterns. Nevertheless, these non-canonical or signaling-related functions should be interpreted in an isoform-specific and context-dependent manner rather than generalized to all Prdx family members [25,26,27].

In this review, we discuss the roles of Prdxs in the nervous system with emphasis on isoform-specific mechanisms. We first summarize how Prdx isoforms contribute to neuronal development and differentiation, including GDE2-dependent motor neuron differentiation. We then discuss their compartment-specific roles in mitochondrial and ER redox homeostasis, followed by their involvement in major neurological disorders, including Alzheimer’s disease, Parkinson’s disease, and ischemic stroke. By integrating these perspectives, this review aims to clarify how individual Prdx isoforms regulate neural physiology and pathology through distinct cell-type- and organelle-dependent mechanisms.

Mammalian Prdx isoforms differ in catalytic class, peroxide-reducing mechanism, subcellular localization, and reported neural cell-type distribution. These isoform-specific features provide a basis for interpreting their distinct roles in neuronal development, organelle homeostasis, and disease-related responses.

## 2. Roles of Peroxiredoxins in Neuronal Development

Prdx isoforms contribute to neuronal development by regulating redox-sensitive signaling events that control progenitor maintenance, cell-cycle exit, and lineage progression [28,29]. Rather than acting only as general peroxide-removing enzymes, specific Prdx isoforms influence developmental signaling through mechanisms that include local H_2_O_2_ control, thiol-disulfide exchange, and modulation of protein activity [18,30,,31]. In this context, several Prdx isoforms participate in peroxide reduction, their functions in the nervous system are not limited to oxidant detoxification and may also include isoform-specific roles in neurogenesis and neuronal development [30,32,,33]. Because these functions differ according to isoform, development stage, and experimental model, representative studies linking Prdxs to neuronal differentiation and neural development are summarized in Table 2.

### 2.1. Redox Control in Neuronal Differentiation

Prdx1 may influence neuronal differentiation by regulating the redox threshold required for differentiation-associated signaling [29]. Epolactaene (Epo), isolated from *penicillium sp.*, and its derivative Epo-C12 have been reported to induced neurite outgrowth and neuronal differentiation in neuroblastoma cell. In this context, Epo-C12 binds cysteine residues in Prdx1, and selectively inhibiting its peroxidase activity without suppressing its chaperone functions [34]. As this interaction has thus far been demonstrated only in a leukemia cell model, the direct contribution of Prdx1 inhibition in neuronal differentiation remains to be established. However, in this model, inhibition of Prdx1 increased intracellular ROS levels, which were associated with enhanced neuronal differentiation [30,33,35]. These findings suggest that appropriate increase in ROS accumulation is required to activate differentiation-related signaling pathways and that the partial suppression of Prdx1 may shift the intracellular redox state in a manner that promotes differentiation. Thus, Prdx1 appears to act as a redox-dependent regulator that sets a threshold for neuronal differentiation, and its inhibition can further facilitate neurite outgrowth by modulating intracellular redox status.

Prdx6 appears to be predominantly expressed in astrocytes in the CNS, with only very low neuronal expression reported and no convincing detection in microglia or oligodendrocytes. Accordingly, in the absence of direct evidence for neuronal Prdx6 expression or neuron-autonomous Prdx6 action, its effects on neurons should be interpreted primarily as indirect, astrocyte-mediated effects [19,20,32]. Prdx6 has been identified as a negative regulator of neurogenesis in neural stem cells [36]. Overexpression of Prdx6 significantly reduced neural differentiation capacity, and this effect was accompanied by decreased expression of neuronal differentiation markers, including NeuN and MAP2 [36,37]. Mechanistically, Prdx6 overexpression led to a markedly downregulated the expression of WDFY1, a key adaptor protein involved in TLR4 signaling, which is known to regulate neural precursor cell differentiation. Suppressing the WDFY1-TLR4 pathway impaired neural stem cell differentiation, indicating that Prdx6 inhibits neurogenesis by modulating this signaling axis [36]. However, current evidence does not establish a direct physical interaction between Prdx6 and WDFY1. Therefore, Prdx6 should be interpreted as a regulator of an intermediary WDFY1-TLR4 signaling pathway rather than as a confirmed direct binding partner of WDFY1. This finding suggests that, in addition to regulating redox balance, Prdx6 functions as a signaling modulator that interacts with specific molecular pathways to control neural stem cell fate.

Beyond their enzymatic antioxidant functions, Prdxs may also influence neuronal differentiation through structural properties unrelated to their canonical peroxidase activity. A mutant form of Prdx1 (Prdx1-C48S), which lacks peroxidase activity, was shown to self-assemble into nanotube-like structures [30,38]. This mutant Prdx1-derived scaffold supported SH-SY5Y cell growth and neuronal differentiation in the absence of conventional differentiation supplements. This effect was accompanied by increased expression of neuronal markers, including β-tubulin III and NF-200, whereas wild-type Prdx1 did not elicit similar activity [38]. Since differentiation was achieved through an engineered Prdx-based scaffold rather than endogenous Prdx signaling, these findings could be interpreted cautiously. Nevertheless, they provide an example in which a Prdx-derived structure can influence neuronal phenotype acquisition through a mechanism distinct from canonical peroxide scavenging.

### 2.2. Redox Control Regulation of Motor Neuron Differentiation via GDE2

Prdx1 and Prdx4 regulate motor neuron differentiation by controlling the redox state and surface availability of glycerophosphodiester phosphodiesterase 2 (GDE2), a key pro-neurogenic transmembrane protein [30,33,39,40,41]. Glycerophosphodiester phosphodiesterase 2 (GDE2) induces motor neuron differentiation by antagonizing Notch signaling, a pathway that maintains progenitor cell identity [42,43]. Activation of GDE2 promotes cell cycle exit and neuronal differentiation, highlighting its critical role in neurogenesis [43,44]. Since GDE2 activity depends on the redox state of its cysteine residues, Prdx-dependent regulation of intracellular and extracellular redox balance is essential for proper motor neuron development.

Prdx4, an endoplasmic reticulum (ER)-localized peroxidase, functions as a compartment-specific redox sensor that regulates the progression of motor neuron differentiation [31,45]. In response to fluctuations in ER-localized H_2_O_2_, Prdx4 modulates the trafficking of GDE2 to the plasma membrane [46]. Mechanistically, Prdx4 promotes oxidation of extracellular cysteine residues in GDE2, thereby limiting its surface expression [46]. As a result, GDE2-mediated inhibition of Notch signaling is reduced, delaying motor neuron differentiation. Experimental evidence has demonstrated that increased Prdx4 activity suppresses GDE2 surface localization and delays neurogenesis, whereas reduced activity enhances GDE2 trafficking and accelerates differentiation. This pattern indicates that Prdx4 is not merely a general antioxidant enzyme but a redox-dependent regulator that controls the temporal dynamics of neurogenesis by regulating GDE2 availability at the cell surface.

In contrast, Prdx1 positively regulates motor neuron differentiation by modulating GDE2 activity. Prdx1 and GDE2 cooperate in the regulation of motor neuron differentiation, and loss of Prdx1 produces defects similar to GDE2 deficiency. Mechanistically, Prdx1 activates GDE2 by reducing an intramolecular disulfide bond, indicating that Prdx1 directly regulates a defined pro-neurogenic protein target rather than simply scavenging excess peroxides [33,47]. This mechanism directly supports the idea that Prdxs can influence neuronal development through specific protein regulation and thiol-disulfide exchange.

These studies demonstrate that Prdxs regulate motor neuron differentiation through opposing, redox-dependent mechanisms of over GDE2 function. While Prdx4 suppresses motor neuron differentiation by restricting GDE2 trafficking to the cell membrane, Prdx1 promotes differentiation by directly activating GDE2 through thiol-disulfide regulation. This reciprocal regulation highlights a redox sensitive developmental mechanism in which oxidation and reduction of the same target protein dynamically control the timing and progression of neurogenesis. Collectively, studies on neuronal differentiation and organelle homeostasis indicate that Prdx isoforms act through distinct mechanisms depending on their catalytic properties and subcellular localization (Table 2).

**Table 2:** Summary of Prdx-related studies in neuronal development and organelle homeostasis.

Isoform	Biological Context	Model System	Main Outcome	Interpretation	Key Reference
Prdx1	Neuronal differentiation	Embryonic stem cell-derived neurogenesis model	Prdx/JNK axis regulated stemness and neuronal differentiation	Prdx1 contributes to redox-sensitive neurogenesis	Kim et al., 2014 [30]
Prdx1	Motor neuron differentiation	Developing motor neuron system	Prdx1 activated GDE2 through thiol-redox regulation	Prdx1 promotes motor neuron differentiation.	Yan et al., 2009 [33]
Prdx1 mutant C48S	Neuronal differentiation	SH-SY5Y cells	Prdx-derived scaffold supported neuronal marker expression	Engineered Prdx structure may support neuronal phenotype acquisition	Cimini et al., 2017 [38]
Prdx3	Mitochondrial apoptosis signaling	Receptor-mediated apoptosis model	Prdx3 oxidation occurred early during apoptosis	Prdx3 may participate in mitochondrial apoptotic signaling	Cox et al., 2008 [13]
Prdx3	Traumatic neuronal injury	Traumatic injury model	Prdx3 reduced neuronal apoptosis	Prdx3 protects neurons under traumatic stress	Hu et al., 2018 [48]
Prdx3	Subarachnoid hemorrhage-associated brain injury	Experimental subarachnoid hemorrhage model	Prdx3 improved neuronal survival	Prdx3 has neuroprotective mitochondrial function	Li et al., 2023 [49]
Prdx3/Prdx5	Mitochondrial redox homeostasis	MPP^+^-exposed SH-SY5Y cells	Loss of Prdx3 or Prdx5 increased mitochondrial vulnerability under MPP^+^ stress	Mitochondrial Prdxs support antioxidant defense	De Simoni et al., 2008 [50]
Prdx4	Motor neuron differentiation	Developing spinal motor neuron model	Prdx4 restricted GDE2 surface expression and delayed neurogenesis	Prdx4 negatively regulates motor neuron differentiation timing	Yan et al., 2015 [31]
Prdx4	ER redox homeostasis and oxidative protein folding	Biochemical and ER stress-related models	Oxidized Prdx4 interacted with ER stress and protein-folding proteins	Prdx4 links ER redox state to proteostasis	Elko et al., 2021 [51]
Prdx4	Glutamate-induced neuronal ER stress	Neuronal cell model exposed to glutamate	Prdx4 attenuated glutamate-induced neuronal death and ER stress	Prdx4 protects neurons by reducing ER stress	Kang et al., 2020 [14]
Prdx4	Learning and memory-related ER stress	Prdx4-deficient mice	Prdx4 deficiency is associated with increased brain ER stress	Prdx4 supports ER homeostasis and cognitive function	Homma et al., 2022 [52]
Prdx5	Mitochondria–ER communication	MPP^+^-induced neuronal cell death model	Prdx5 is linked to mitochondrial redox balance	Prdx5 may modulate mitochondria–ER crosstalk	De Simoni et al., 2013 [53]
Prdx6	Neural stem cell differentiation	Neural stem cells	Prdx6 overexpression suppressed neurogenesis through WDFY1–TLR4 signaling	Prdx6 acts as a negative regulator of neurogenesis	Yeo et al., 2019 [36]
Prdxs and Trxs	Embryonic spinal cord development	Mouse embryonic spinal cord	Prdx and Trx isoforms showed stage- and region-specific expression	Redox proteins may regulate spinal cord development	Pirson and Knoops, 2015 [46]

This table summarizes representative studies linking Prdx isoforms to neuronal differentiation, mitochondrial redox regulation, ER homeostasis, and mitochondria–ER communication. The studies indicate that Prdxs act in an isoform- and compartment-dependent manner rather than functioning as interchangeable peroxide-removing enzymes.

## 3. Roles of Peroxiredoxins in Organelle Redox Homeostasis

Prdx-mediated organelle redox regulation is a central component of neuronal homeostasis, particularly in mitochondria and the endoplasmic reticulum (ER) [54,55]. As summarized in Table 2, mitochondrial Prdx3 and Prdx5 are closely associated with mitochondrial peroxide metabolism, preservation of mitochondrial membrane potential, and suppression of mitochondria-mediated apoptosis, whereas ER-localized Prdx4 links peroxide metabolism to oxidative protein folding and ER proteostasis [13,14,49,50,51,52,53]. These compartment-specific functions are especially important in neurons, where ATP production, calcium flux, protein folding, and stress adaptation must be tightly coordinated [13,48,49,50,53].

### 3.1. Prdx3 and Prdx5 in Mitochondrial Homeostasis

Mitochondria are a major source of intracellular reactive oxygen species (ROS), and mitochondrial redox balance regulation is essential for cell survival [56,57]. Mitochondrial Prdxs, particularly Prdx3 and Prdx5, play critical roles in controlling ROS levels and preserving mitochondrial functional integrity. In SH-SY5Y cells, silencing of Prdx3 or Prdx5 does not significantly affect basal mitochondrial proliferation but markedly reduces mitochondrial antioxidant capacity, and under oxidative stress induced by the mitochondrial complex I inhibitor MPP+, Prdx3/5-deficient cells experience increased protein oxidation and apoptotic cell death [50]. These findings indicate that both Prdx3 and Prdx5 contribute to mitochondrial peroxide control under neurotoxic stress conditions. However, the available evidence for Prdx5 in neuronal mitochondrial homeostasis is more limited than that for Prdx3. In particular, Prdx5 has mainly been examined in SH-SY5Y cell-based MPP+ models, where its loss increases oxidative vulnerability [53,58], whereas fewer in vivo studies have directly tested Prdx5-specific mitochondrial protection in neuronal injury models [59,60]. Prdx3’s protective role is further demonstrated by injury models in which mitochondrial dysfunction is a prominent pathogenic feature. In primary cortical neurons subjected to traumatic injury and subarachnoid hemorrhage, Prdx3 overexpression preserved mitochondrial membrane potential and ATP production and suppressed mitochondria-mediated apoptosis. In subarachnoid hemorrhage mammal models, Prdx3 consistently improved neuronal survival and behavioral characteristics. These studies support the interpretation that Prdx3 functions not only as a peroxide-detoxifying enzyme but also as a stabilizer of mitochondrial homeostasis during periods of neuronal stress [48,49].

In addition to its role as an antioxidant, Prdx3 may also participate in mitochondrial redox signaling during apoptosis. Earlier work showed that during receptor-mediated apoptosis, Prdx3 undergoes selective oxidation during an early phase, preceding more global oxidative disruption, with its timing correlating with caspase activation and cytochrome c release. This observation suggests that Prdx3 is not merely a passive ROS scavenger but may also function as a redox-sensitive component of the mitochondrial apoptotic response [13].

### 3.2. Prdx4 in ER Redox Homeostasis

The endoplasmic reticulum is a specialized oxidative compartment in which protein folding, disulfide bond formation, and redox control are tightly interconnected [61,62,63,64]. As the only Prdx localized to the ER lumen, Prdx4 performs function central to ER function by coupling peroxide detoxification and oxidative protein folding. Biochemical studies have shown that Prdx4 can use hydrogen peroxide to oxidize protein disulfide isomerases, supporting proper disulfide bond formation while simultaneously removing ROS [51].

Disruption of Prdx4 function leads to increased ER stress, alterations in ER-associated degradation, and the dysregulation of calcium homeostasis. In Prdx4-deficient mice, increased ER stress in the brain has been associated with deficits in spatial learning and memory, despite an absence of major gross structural abnormalities [52]. During periods of high oxidative stress, Prdx4 also appears to limit ER stress-associated apoptosis, and in neuronal models exposed to glutamate or ROS, increased Prdx4 activity has been associated with improved cell survival and the reduced expression of ER stress markers such as BIP/GRP78, CHOP, and caspase-12. Although the exact contributions of Prdx4 may vary depending on the model system, the overall evidence suggests it is a key regulator linking ER redox homeostasis, protein quality control, and stress resistance [14].

### 3.3. Prdx5 in Mitochondria–ER Crosstalk

In addition to its antioxidant role within mitochondria, Prdx5 has recently been proposed that Prdx5 is a modulator of mitochondria–ER crosstalk through mitochondria-associated membranes. Altered Prdx5 expression has been associated with changes in both redox status and calcium-related signaling across the mitochondria–ER axis. Loss of Prdx5 exacerbates the oxidative vulnerability of the system, possibly leading to a coordinated dysfunction involving mitochondrial ROS accumulation and ER stress-related signaling, whereas Prdx5 overexpression has been linked to improved protection against oxidative injury and organelle stress. However, direct evidence identifying Prdx5 as a structural regulator of mitochondria-associated membranes remains limited [53].

Collectively, prior research indicates that Prdxs are integral components of neuronal homeostasis at the organelle level. Together, Prdx3 and Prdx5 preserve mitochondrial redox balance and stress resilience, whereas Prdx4 supports ER oxidative folding and proteostasis. Additionally, Prdx5 may further contribute to the functional coupling of mitochondria and the ER. Thus, individual Prdx isoforms do not function as interchangeable ROS scavengers; they are compartment-specific regulators that stabilize neuronal physiology through distinct yet partially overlapping mechanisms. Understanding this framework provides an important basis for deciphering how disruptions in Prdx-dependent homeostasis may contribute to neuronal injury and neurodegenerative disease [48,49,50,51,52,53,65].

## 4. Peroxiredoxins in Disease Pathology

Prdx functions in neurological disease models are highly context-dependent and cannot be interpreted solely on the basis of antioxidant capacity [10]. Depending on the iso-form, cell type, subcellular localization, disease stage, and extracellular release, Prdxs may contribute to neuronal protection, glial inflammatory signaling, or damage-associated molecular pattern-like activity [66]. To facilitate comparison across disease models, representative studies on Prdx isoforms in Alzheimer’s disease, Parkinson’s disease, ischemic stroke, and extracellular inflammatory signaling are summarized in Table 3.

This table summarizes representative studies examining Prdx isoforms in Alzheimer’s disease-related models, Parkinson’s disease-related models, ischemic stroke, and extracellular inflammatory signaling. The indicates that Prdx isoforms are associated with each disease and the function depends on isoform, cell type, and localization.

### 4.1. Alzheimer’s Disease

In Alzheimer’s disease-related models, Prdx1, Prdx5, and Prdx6 have been implicated in distinct aspects of amyloid-β toxicity, mitochondrial dysfunction, axonal transport impairment, and glial responses, as summarized in Table 3. These findings indicate that Prdx function in AD-related pathology is isoform- and cell-type-dependent rather than uniformly protective or detrimental. In particular, Prdx-dependent microglial activation appears to be mediated through extracellular DAMP-like signaling or glia–glia communication pathways, rather than through a single uniform mechanism.

Among Prdx isoforms, Prdx5 has consistently been reported to exert protective effects in AD models. Prdx5 expression is significantly increased after amyloid-β oligomer exposure, suggesting the induction of a compensatory antioxidant response under pathological stress [69]. Functionally, Prdx5 overexpression reduces ROS accumulation and suppresses Ca^2+^-dependent calpain activation, thereby preventing neuronal death and abnormal Cdk5 activation [69]. In addition, it attenuates ERK–Drp1-mediated mitochondrial fragmentation, contributing to the maintenance of mitochondrial dynamics and neuronal survival under amyloid-β-induced stress [70]. Based on these findings Prdx5 appear to be a stress-inducible protective factor in AD-related pathologies.

Prdx1 also exerts neuroprotective effects under the streptozotocin-induced AD-like condition by reducing oxidative stress and blocking the Ca^2+^–calpain–Cdk5 signaling pathway, thereby preserving mitochondrial integrity and increasing neuronal survival [67]. In addition, the increased acetylation of Prdx1 has been reported to alleviate amyloid-β-induced impairment of axonal transport, highlighting the importance of Prdx functional regulation in the pathological AD environment [68].

In Alzheimer’s disease models, Prdx6 has been reported to exert protective effects in several contexts, including improvement of memory dysfunction, attenuation of oxidative stress and amyloid-β-accumulation, neuroprotection against tau toxicity, and reduction in Aβ pathology [20]. These observations indicate that the effect of Prdx6 is context-dependent and varies across experimental systems, rather than supporting a simple conclusion that Prdx6 is generally detrimental [32]. However, not all Prdx isoforms exert protective effects in disease conditions. Prdx6 is predominantly expressed in astrocytes, and its expression is altered in the vicinity of amyloid plaques, suggesting its involvement in oxidative stress responses associated with AD pathology [19]. One study reported that Prdx6 overexpression aggravated AD-like changes in a subacute Aβ oligomer infusion model [77]. In contrast, astrocytic Prdx6 has been shown to facilitate plaque-associated microglial responses and attenuate amyloid pathology in APP/PS1 mice with modest Prdx6 overexpression [20]. Cell culture studies also suggest a protective role of Prdx6 against Aβ oligomer-induced toxicity [32]. These findings may reflect differences in experimental design, including acute Aβ oligomer exposure versus chronic plaque formation, the cellular source and expression level of Prdx6, and the presence or absence of loss-of-function validation [20,32]. In this regard, the APP/PS1-based study with Prdx6 haplodeficiency provides stronger genetic support for an astrocyte-associated protective role of Prdx6 in chronic amyloid pathology [20,77], whereas the Aβ oligomer infusion study should be interpreted with caution because it lacks complementary loss-of-function validation [77].

In addition, associations between dentate gyrus Prdx6 levels and spatial memory impairment have been reported primarily in aging-related memory paradigms rather than as an AD-specific mechanism [78]. Therefore, Prdx6 should be described not as a pathogenic factor, but rather as a glial regulator whose role in AD varies with cellular and pathological conditions. Although earlier studies tended to place Prdx6 within broader AD-associated proteomic signatures and cross-platform replication frameworks rather than explicitly emphasizing its upregulation in AD, subsequent evidence has more directly supported this interpretation [19]. Notably, a recent proteomic meta-analysis identified Prdx6 as one of the core proteins upregulated in Alzheimer’s disease based on integrated CSF and brain proteome datasets, and more recent bioinformatic and experimental analyses further demonstrated significant Prdx6 upregulation in the entorhinal cortex in AD [79,80,81]. Accumulating evidence suggests that Prdx6 may exert context-dependent, and at times apparently opposing, effects in CNS disorders. This duality is most clearly illustrated in Alzheimer’s disease, where Prdx6 has been reported to attenuate Aβ pathology through astrocyte-associated protective mechanisms in some models, whereas in other experimental paradigms its overexpression aggravated amyloid-β-accumulation, oxidative stress, and cognitive dysfunction [20].

### 4.2. Parkinson’s Disease

**Figure 1:**
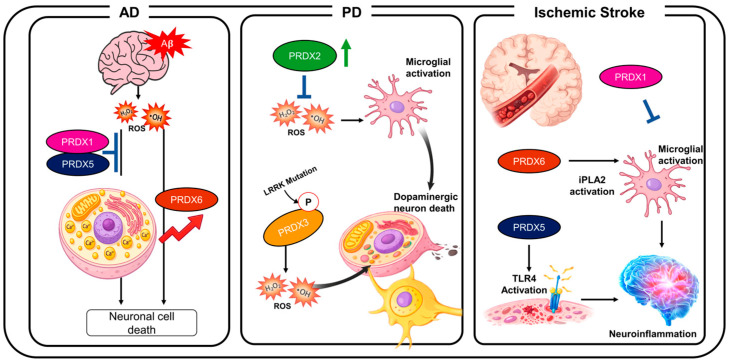
Isoform-specific roles of Prdxs in neurodegeneration and ischemic stroke.

In Parkinson’s disease-related models, Prdx2, Prdx3, and Prdx6 have been examined in relation to oxidative stress, mitochondrial dysfunction, dopaminergic neurodegeneration, and glial inflammatory responses (Table 3). Although Prdx2 and Prdx3 are generally associated with protective antioxidant or mitochondrial functions, Prdx6 has been reported to exacerbate dopaminergic neurodegeneration in an MPTP model, emphasizing the isoform-specific nature of Prdx biology in PD [71,72]. In PD animal models, increased ROS is a major contributor to mitochondrial damage and neuronal cell death. Specifically, excessive ROS accumulation promotes dopaminergic neuron degeneration, making it a key pathological mechanism driving disease progression [72]. Under these pathological conditions, Prdx family proteins function as important regulators of antioxidant responses [71]. For example, Prdx2 is reported to play a protective role in cellular PD models. In MPP^+^-treated SH-SY5Y cells, Prdx2 overexpression reduces oxidative stress and, thus, attenuates neuronal toxicity, improving survival and preserving healthy dopaminergic markers (Figure 1). These findings support a view in which Prdx2 is an important antioxidant in the cellular defense against PD-related oxidative injury rather than a passive marker of redox imbalance [71].

In PD models, Prdx3 provides a disease-relevant example of how mitochondrial Prdx function can be altered by pathological signaling. Prdx3 overexpression or gene delivery has been reported to attenuate dopaminergic neurodegeneration [72,74], whereas LRRK2-associated phosphorylation of Prdx3 impairs its antioxidant activity and increases oxidative vulnerability [73]. However, mutations in leucine-rich repeat kinase 2 (LRRK2), a representative genetic factor associated with PD, increase the phosphorylation of Prdx3, which impairs its antioxidant function, in turn exacerbating oxidative stress and, ultimately promoting neuronal cell death (Figure 1). These findings indicate that Prdx3 function is regulated through not only expression level but also post-translational modification [73]. Furthermore, they suggest that thiol peroxidase family proteins contribute to the maintenance of mitochondrial homeostasis [72].

Taken together, these studies demonstrate that Prdxs function as dynamic regulators whose activity is modulated by intracellular redox states and signaling pathways, highlighting their critical role in the progression of PD [71,72,73,74]. This further suggests that targeting mitochondrial function and redox balance may be successful therapeutic strategies for disease intervention.

### 4.3. Ischemic Stroke

In ischemic stroke, Prdx isoforms show a clear compartment-dependent duality, with intracellular Prdxs contributing to cytoprotection or microglial state regulation, whereas extracellular Prdxs can function as damage-associated molecular patterns that amplify post-ischemic inflammation (Table 3). Prdxs show a distinct duality in ischemic stroke: they can contribute to intracellular protection, yet once released from injured cells, they may act as damage-associated molecular patterns (DAMPs) in the extracellular space, amplifying post-ischemic inflammation [10,76]. Experimental stroke studies have identified Prdx family proteins, including Prdx1, Prdx2, Prdx5, and Prdx6, as extracellular DAMP-like mediators that activate innate immune pathways such as TLR2 and TLR4 signaling [66,76]. In addition, mechanistic studies have shown that extracellular Prdx5 can activate TLR4 in a redox-state-dependent manner, although this evidence was obtained outside the stroke model context [57].

Isoform-specific functions of Prdx proteins have also been observed in ischemic stroke. Prdx 1 acts as an antioxidant enzyme, playing a protective role by regulating stroke-associated microglial states and limiting excessive inflammatory responses in acute ischemic injury sites. This suggests that Prdx1’s ROS-scavenging activity contributes to reducing neuronal damage [69,75]. In contrast, Prdx6 has been shown to promote inflammatory responses following ischemic stroke. This isoform induces M1 phenotype microglial activation through its iPLA2-associated function in astrocytes, thereby increasing neuroinflammation and exacerbating brain injury [65]. Mechanistically, Prdx6-associated iPLA2 activity in astrocytes appears to enhance astrocytic inflammatory signaling, which subsequently promotes the polarization of neighboring microglia toward an M1-like pro-inflammatory phenotype. Thus, Prdx6-dependent microglial activation in ischemic stroke is best interpreted as an indirect astrocyte–microglia communication pathway rather than a direct microglia-intrinsic action of Prdx6.

Although intracellular Prdx5 is generally considered protective, extracellular Prdx5 can interact with TLR4 and exhibit DAMP-like proinflammatory activity in a redox-state-dependent manner. Current understanding of extra cellular Prdx function is derived from mechanistic studies of extracellular Prdx4-TLR4 signaling rather than on stroke models [66]. Therefore, it is more appropriate to consider Prdx5 to be a potential redox-dependent mediator of post-ischemic inflammation than a driver of stroke pathology.

Ischemic stroke research has characterized Prdxs as possessing dual compartment-dependent functions, acting not only as intracellular antioxidant enzymes but also, under certain conditions, as extracellular mediators of inflammation [10,76]. This duality makes Prdx-centered pathways attractive but complex therapeutic targets, since effective intervention may require the preservation of protective intracellular functions while limiting harmful extracellular inflammatory signaling (Figure 1).

### 4.4. Post-Translational Regulation and Functional Switching of Prdxs

The functional diversity of Prdxs is further shaped by redox-dependent structural changes and post-translational modification, which can influence their catalytic activity, subcellular behavior, oligomeric state, and protein interactions [9,51]. Under Physiological conditions or moderate oxidative stress, Prdxs primarily function as peroxidases that reduce H_2_O_2_ and organic peroxides through conserved catalytic cysteine residues, thereby contributing to peroxide detoxification and redox signaling control [9]. When exposed to sustained oxidative stress, however, oxidation of Prdxs can modify their oligomeric state and interactions with stress-related proteins, suggesting that Prdx function may shift from peroxide removal toward chaperone-like or proteostasis-related functions [9,51]. This concept is supported by evidence that oxidation of Prdx4 induces oligomerization and promotes interactions with proteins involved in protein folding and ER stress, linking the Prdx redox state to proteostasis regulation [51]. In addition to redox-dependent oxidation, phosphorylation can modify Prdx activity under disease-related conditions. In Parkinson’s disease-related models, LRRK2-associated phosphorylation of Prdx3 has been reported to impair Prdx3 function and increase oxidative stress-induced neuronal vulnerability [73]. Acetylation may also regulate Prdx function in neurons. In an amyloid-β model, increased acetylation of Prdx1 following HDAC6 inhibition was associated with recovery of impaired axonal transport, suggesting that Prdx acetylation can influence neuronal function beyond peroxide scavenging [68]. Although other cysteine-based modifications may also contribute to Prdx regulation, their isoform-specific roles in neuronal Prdx biology remain less clearly defined. Therefore, Prdxs may be better understood as redox-responsive molecular switches rather than static antioxidant enzymes. Their functional output may depend on peroxide burden, redox state, post-translational modification, oligomerization, subcellular localization, and cell-type-specific context [9,10,16,51].

Prdxs exhibit distinct, isoform-dependent functions across multiple neurological conditions. In Alzheimer’s disease (AD), Prdx1 and Prdx5 attenuate ROS accumulation, whereas Prdx6 is associated with increased oxidative stress and neuronal death. In Parkinson’s disease (PD), Prdx2 limits ROS and microglial activation, while Prdx3 dysfunction contributes to oxidative damage and dopaminergic neuron loss. In ischemic stroke, Prdx1 exerts protective effects, whereas Prdx6 promotes microglial activation via iPLA2 and when released to the extracellular space, Prdx5 drives TLR4-mediated neuroinflammation.

## 5. Conclusions

The recent studies discussed in this review indicate that in the brain, Prdxs serve roles beyond those of simple antioxidant enzymes. They function as isoform-specific regulators of redox-dependent signaling across multiple levels of neural biology, from neuronal differentiation to organelle homeostasis to disease-associated stress response [18]. During development, Prdxs help the progression of neuronal differentiation by modulating redox-sensitive pathways [82]. In mature neural cells, they contribute to mitochondrial integrity, ER proteostasis, and communication between intracellular compartments, thereby supporting neuronal stability under both physiological and stress conditions [16,51]. Under pathological condition, however, their roles become more complex, being strongly dependent on isoform and localization, and being cell-type specific [83]. While several Prdxs are associated with cytoprotective functions, the same isoforms or others may participate in inflammatory or maladaptive responses under pathological conditions [66]. These findings make clear that Prdxs in the nervous system do not merely function as peroxide scavengers but have the capacity to precisely regulate redox signaling.

At the same time, the current research suggests that our understanding of Prdx biology in the brain is still evolving. Many findings have been obtained from acute stress models or experimental systems that do not fully capture the diversity of neural cell types and disease stages in vivo. In addition, the factors that determine whether a given Prdx response becomes protective or detrimental particularly those related to subcellular localization, extracellular release, and post-translational regulation remain incompletely understood. Further clarification of these mechanisms will require cell-type-specific and compartment-resolved approaches, together with direct analyses of Prdx redox states and modifications in the intact nervous system. Such studies should help clarify how Prdx-dependent pathways are integrated across development, homeostasis, and disease, and whether they can be targeted therapeutically without interfering with their essential physiological functions.

## Figures and Tables

**Table 1:** Isoform-specific biochemical properties, subcellular localization, and neural distribution of mammalian Prdxs.

Isoform	Biochemical Class	Key Catalytic Feature	Major Subcellular Localization	Distribution in the CNS/Neural Cell Types
P rdx1	Typical 2-Cys Prdx	Thioredoxin-dependent peroxidase	Mainly cytosol; reported in nucleus	Reported in neuronal populations and neural progenitor-related systems
			depending on cell state	
Prdx2	Typical 2-Cys Prdx	abundant peroxide-reducing enzyme	Mainly cytosol	Expressed in the brain, with reported neuronal
				distribution in several regions
Prdx3	Typical 2-Cys Prdx	Specialized mitochondrial peroxide detoxification	Mitochondria	Expressed in neural cells with prominent relevance to
				mitochondrial redox regulation
Prdx4	Typical 2-Cys Prdx	Secretory pathway/ER-associated peroxide metabolism	Endoplasmic reticulum	Reported in the nervous system, including developing neural tissues
Prdx5	Atypical 2-Cys Prdx	compartment-specific redox regulation	Mitochondria, peroxisomes, cytosol	Expressed in neural cells; functionally linked to mitochondrial protection and mitochondria–ER
				communication
Prdx6	1-Cys Prdx	Unique dual function as phospholipid hydroperoxide peroxidase and iPLA2-realted enzyme	Cytosol, lysosome-related compartments,	Predominantly associated with astrocytes in the CNS;
			membrane-associated contexts	neuronal effects are often interpreted as indirect or glia-mediated

**Table 3:** Summary of Prdx-related studies in neurological disease models.

Pathological Context	Prdx Isoform	Key References	Main Outcome	Interpretation	Model System
**Alzheimer’s Disease**	Prdx1	Park et al., 2024 [67]	Reduced mitochondrial fragmentation and	Protective stress-response function	Streptozotocin-induced AD-like hippocampal neuronal model
			neuronal injury		
		Choi et al., 2017 [68]	Restored impaired axonal transport	PTM-dependent protective function	Amyloid-β-induced
					axonal transport dysfunction
	Prdx5	Park et al., 2017 [69]	Reduced neuronal death signaling	Protective neuronal response	AβO-treated neuronal cells
		Kim et al., 2016 [70]	Recued mitochondrial fragmentation	Protective mitochondrial dynamics	
	Prdx6	Power et al., 2008 [19]	Association with AD pathology	Glia-dependent role	Human AD brain tissue
**Parkinson’s Disease**	Prdx2	Liu et al., 2023 [71]	Reduced oxidative stress and toxicity	Protective cytosolic response	MPP+-treated SH-SY5Y cells
	Prdx3	Angeles et al., 2014 [72]	Reduced mitochondrial and	Protective mitochondrial redox regulation	Drosophila LRRK2 mutant model
			dopaminergic degeneration		
		Angeles et al., 2011 [73]	Increased oxidative vulnerability	PTM-linked loss of Prdx3 function	LRRK2 mutation-associated
					neuronal model
		Villa-Cedillo et al., 2025 [74]	Reduced neurodegeneration	Protective mitochondrial intervention	Animal model of PD
	Prdx6	Yun et al., 2015 [37]	Worsened dopaminergic degeneration	Potentially detrimental, model-dependent	MPTP mouse model
**Ischemic stroke**	Prdx1	Kim et al., 2022 [75]	Protective cell-state regulation	Reduced acute ischemic injury	Acute ischemic stroke model
	Prdx family	Shichita et al., 2012 [76]	Initiated post-ischemic inflammation	Extracellular pro-inflammatory function	Experimental ischemic
					stroke model
**Neuroinflammation**	Prdx6	Peng et al., 2024 [65]	Increased M1 microglial activation	Glia-mediated inflammatory mechanism	Astrocyte-microglia stroke model

## Data Availability

No new data were generated and analyzed in this study. All schematic figures were created by the authors based on the cited literature.

## Abbreviations

The following abbreviations are used in this manuscript:

ROS	Reactive oxygen species
Prdxs	Peroxiredoxins
ER	Endoplasmic reticulum
Epo	Epolactaene
GDE2	Glycerophosphodiester phosphodiesterase 2
AD	Alzheimer’s disease
Cdk5	Cyclin-dependent kinase 5
PD	Parkinson’s disease
LRRK2	Leucine-rich repeat kinase 2
DAMP	Damage-associated molecular patterns
iPLA2	Calcium-independent phospholipase A2
